# Amorphization of Thiamine Chloride Hydrochloride: Effects of Physical State and Polymer Type on the Chemical Stability of Thiamine in Solid Dispersions

**DOI:** 10.3390/ijms21165935

**Published:** 2020-08-18

**Authors:** Seda Arioglu-Tuncil, Adrienne L. Voelker, Lynne S. Taylor, Lisa J. Mauer

**Affiliations:** 1 Department of Food Science, Purdue University, 745 Agriculture Mall Drive, West Lafayette, IN 47907, USA; starioglu@beu.edu.tr (S.A.-T.); avoelke@purdue.edu (A.L.V.); 2 Department of Industrial and Physical Pharmacy, Purdue University, 575 Stadium Mall Drive, West Lafayette, IN 47907, USA; lstaylor@purdue.edu

**Keywords:** vitamin B_1_, thiamine, thiamine chloride hydrochloride, thiamine degradation, amorphous solid dispersion, chemical stability, chemical degradation, amorphous stability

## Abstract

Thiamine is an essential micronutrient, but delivery of the vitamin in supplements or foods is challenging because it is unstable under heat, alkaline pH, and processing/storage conditions. Although distributed as a crystalline ingredient, thiamine chloride hydrochloride (TClHCl) likely exists in the amorphous state, specifically in supplements. Amorphous solids are generally less chemically stable than their crystalline counterparts, which is an unexplored area related to thiamine delivery. The objective of this study was to document thiamine degradation in the amorphous state. TClHCl:polymer dispersions were prepared by lyophilizing solutions containing TClHCl and amorphous polymers (pectin and PVP (poly[vinylpyrrolidone])). Samples were stored in controlled temperature (30–60 °C) and relative humidity (11%) environments for 8 weeks and monitored periodically by X-ray diffraction (to document physical state) and HPLC (to quantify degradation). Moisture sorption, glass transition temperature (T_g_), intermolecular interactions, and pH were also determined. Thiamine was more labile in the amorphous state than the crystalline state and when present in lower proportions in amorphous polymer dispersions, despite increasing T_g_ values. Thiamine was more stable in pectin dispersions than PVP dispersions, attributed to differences in presence and extent of intermolecular interactions between TClHCl and pectin. The results of this study can be used to control thiamine degradation in food products and supplements to improve thiamine delivery and decrease rate of deficiency.

## 1. Introduction

Thiamine (vitamin B_1_), the first vitamin to be discovered, is an essential nutrient that is involved in many physiological activities in the human body, including energy metabolism; synthesis of several neurotransmitters; and tissue, brain, and organ functions [1]. As a result of being a water-soluble vitamin, thiamine is held in reserve in the body only for 2–3 weeks [1]; therefore, continuous dietary intake of thiamine is important for maintaining health. The recommended daily thiamine intake is 1.2 mg [2]. When considering the physiological functions of thiamine, it is not surprising that its deficiency results in serious disorders, manifesting within 10 days with symptoms such as irritability, fatigue, weight loss, confusion, and blurred vision, with the potential to lead to the more severe Wernicke–Korsakoff syndrome, beriberi, or Leigh syndrome [3]. Overall rates of thiamine deficiencies in developed countries have been reduced to about 10% due to fortification programs, with greater proportions of alcoholics and people suffering from Celiac disease experiencing thiamine deficiencies [4,5,6]. However, rates of thiamine deficiencies are higher in developing countries, in part due to different dietary habits, with up to 25% of the population experiencing severe deficiency symptoms [6].

Thiamine delivery in the diet can be a challenge because thiamine is one of the most unstable vitamins due to its sensitivity to heat, radiation, alkaline pH, other ingredients (such as salts or sulfites), and processing and storage conditions [7]. The whole food sources of thiamine include yeast, meat (especially pork), whole grains, beans, soybeans, nuts, and egg yolk [3]. Cereal grains are good sources of thiamine; however, since most thiamine is contained in the bran and outer layers of the kernel, most is lost during the refining process in the production of white flour or polished rice [8]. To compensate for this loss of nutrients, a salt form of thiamine is often used for enrichment or fortification, with thiamine chloride hydrochloride (TClHCl) and thiamine mononitrate (TMN) being the most common additive forms, both of which have Generally Recognized as Safe (GRAS) status in the USA (FDA codes: 21CFR184.1875 for chloride hydrochloride and 21CFR184.1878 for mononitrate). TMN is a mono-salt with one nitrate anion present, and TClHCl is a di-salt with two chloride ions present. Both natural and synthetic salt forms of thiamine are known to degrade via oxidation/reduction reactions and interactions with other ingredients [7,9,10]. In addition to loss of vitamin activity, thiamine degradation is also known to produce sulfur-containing degradation products that can contribute significant odors, thereby negatively affecting sensory perception, with one known degradation compound, bis(2-methyl-3-furyl) disulfide, having one of the lowest odor thresholds of any organic compound in water (0.02 parts per trillion) [11,12]; thus, nutritional availability is not the only consideration when monitoring thiamine degradation, even when present in relatively low amounts in food or supplement products.

Although the salt forms of thiamine are distributed in the crystalline form, the amorphous form of small molecules can be produced following interactions with polymers in solid dispersions and/or following some processing methods (e.g., freeze drying, spray drying, milling, grinding) [13,14]. A recent study documented that TClHCl interacts with a variety of polymers, enabling its solidification in the amorphous state when select ratios of thiamine and a polymer are co-lyophilized [15]. Freeze drying is commonly used as an amorphization technique to generate small sample amounts for experimental exploration; it is also used when heat may be harmful to the system, which could lead to degradation in the case of thiamine. Solid state (amorphous vs. crystalline) is known to influence stability, with the chemical reactivity of amorphous solids being higher than that of their crystalline counterparts in many environments, which is often attributed to their greater molecular mobility and hygroscopicity [16]. Despite the possibility that thiamine is present in an amorphous form in some foods and supplements, very few studies have addressed the potential for and/or extent of chemical degradation of thiamine in the amorphous state. Building on our previous study analyzing the amorphization of thiamine using two polymers and the physical stability of those systems [15], the objectives of this study were therefore (1) to document thiamine degradation (monitored as thiamine loss) in a variety of amorphous solid dispersions containing TClHCl and two polymers selected from our previous study and based on their different molecular structures, hydrogen bonding abilities, glass transition temperatures (T_g_), and hygroscopicity traits and (2) to compare the degradation of thiamine in amorphous dispersions to that of crystalline thiamine physically blended with the same polymers. The fundamental understanding of how physical state and intermolecular interactions with other amorphous components affect the chemical stability of amorphous thiamine provides direction to further understand thiamine stability in a variety of food systems and dietary supplements, especially when present in the amorphous state.

## 2. Results and Discussion

### 2.1. Effect of Physical State on Thiamine Degradation

**Figure 1:**
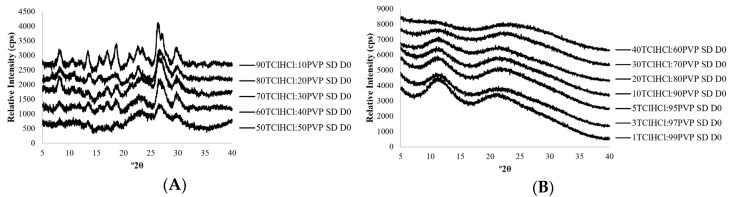
X-ray powder diffraction patterns of TClHCl:PVP solid dispersions (SDs) prepared from (**A**) 50TClHCl:50PVP to 90TClHCl:10PVP, in which all samples exhibited partial crystallinity, and (**B**) 1TClHCl:99PVP to 40TClHCl:60PVP immediately following lyophilization, in which all samples were amorphous.

**Figure 2:**
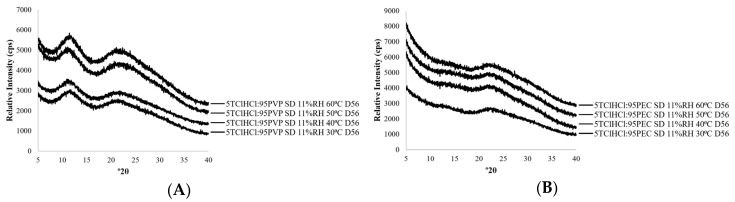
X-ray powder diffraction patterns of (**A**) 5TClHCl:95PVP and (**B**) 5TClHCl:95PEC solid dispersions (SDs) stored at 11% RH and 30–60 °C on day 56.

The physical states of all solid dispersions, before and after storage treatments, were documented using powder x-ray diffraction (PXRD). Consistent with results from a previous study [15], and as shown in Figure 1 and Figure 2, it was possible to stabilize TClHCl in the amorphous form in the presence of both poly(vinylpyrrolidone) (PVP) and pectin (PEC) polymers. A minimum of 60% *w*/*w* PVP was needed to amorphize TClHCl (Figure 1), with less PVP resulting in TClHCl crystallization during or immediately after lyophilization (Figure 1A). While different ratios of PEC dispersions were not prepared in this study, a minimum of only 40% *w*/*w* PEC was needed to amorphize TClHCl [15]. No evidence of thiamine crystallization was found in any of the amorphous vitamin:polymer dispersions, including both PVP and PEC, that were stored at 11% relative humidity (RH) and temperatures ranging from 30–60 °C for 8 weeks (the duration of the chemical stability study) (Figure 2). Therefore, these environmental conditions were chosen to explore differences in the chemical stability of amorphous versus crystalline thiamine.

**Figure 3:**
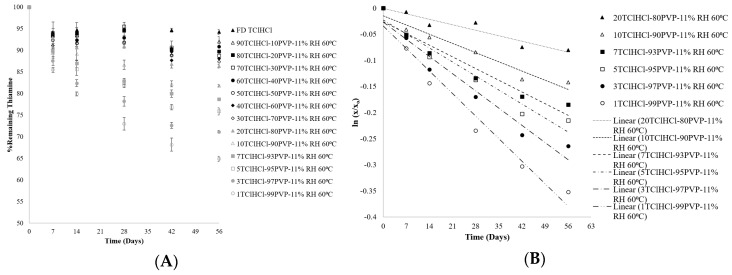
(**A**) Chemical stability of thiamine in various ratios of TClHCl:PVP solid dispersions (SDs) stored at 11% RH and 60 °C for 56 days. (**B**) First-order degradation regression lines of thiamine in various ratios of TClHCl:PVP dispersions stored at 11% RH and 60 **°**C for 56 days.

As shown in Figure 3A, significant differences (*p* < 0.05) in thiamine degradation were found between TClHCl:PVP solid dispersions containing decreasing vitamin:polymer ratios. As the amount of PVP in the solid dispersion increased, thiamine stability decreased. This trend was most evident in dispersions containing 10% or less of thiamine, wherein the percentage of thiamine remaining differed by over 15% between the dispersions with 10% and 1% thiamine after 56 days (82% and 65% thiamine remaining, respectively). Dispersions in which the TClHCl had crystallized (containing ≤ 70% PVP) were the most stable, supporting the hypothesis that crystalline thiamine is more stable than amorphous thiamine. The TClHCl that had been lyophilized in the absence of a polymer was the most stable and was found to have crystallized during or immediately following lyophilization.

In general, thiamine degraded more when amorphous than when crystalline, with degradation rate depending on vitamin proportion in the dispersion (Figure 3). Although completely crystalline thiamine was not expected to degrade over the duration of the study, some degradation was still found in samples in which amorphous thiamine had crystallized. These samples, however, had stability trends in which increasing TClHCl content resulted in less thiamine degradation. Although the percentage of thiamine crystallinity was not quantified in this study, differences in PXRD peak intensities between crystalline samples (Figure 1A) indicated that it was possible that partial crystallization may have been the cause for this observation, with greater amounts of thiamine (and therefore less PVP) resulting in a greater percentage of the less chemically labile crystalline thiamine. That thiamine was most labile and chemically unstable in dispersions containing the lowest vitamin proportions is concerning for foods and supplements, wherein vitamin contents tend to be low in proportion to polymers and other ingredients.

Thiamine degradation has been reported to follow first-order reaction kinetics in foods and solutions [12,17,18], which is consistent with what was found for thiamine degradation in amorphous TClHCl:PVP dispersions in this study (Figure 3B). The observed reaction rate constants (k_obs_) calculated from linear regressions (R^2^ = 0.92−0.96) are reported in Table 1. The k_obs_ values were influenced by the proportion of PVP in the solid dispersion, with k_obs_ increasing as the proportions of PVP increased. The k_obs_ values in this study varied from those in previous studies of reaction kinetics of amorphous thiamine degradation, likely due to different temperature and humidity storage conditions as well as different excipients in the amorphous matrix [17,19,20,21]. The t_90_ values, or time when 90% of thiamine remained (10% thiamine had degraded), ranged from 17 to 70 days, increasing as TClHCl concentration increased from 1 to 20% in the dispersions (Table 1).

### 2.2. Relationship of T−T_g_ with Thiamine Stability

**Figure 4:**
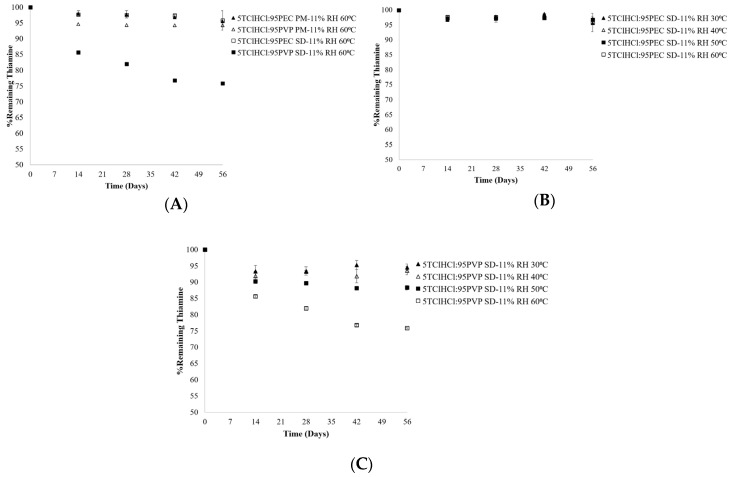
Chemical stability of thiamine in (**A**) 5TClHCl:95PEC and 5TClHCl:95PVP physical mixtures (PMs) compared to solid dispersions (SDs) stored at 11% RH and 60 **°**C for 56 days, (**B**) 5TClHCl:95PEC solid dispersions (SDs) stored at 11% RH and 30–60 **°**C for 56 days, and (**C**) 5TClHCl:95PVP solid dispersions (SDs) stored at 11% RH and 30–60 **°**C for 56 days.

Thiamine was found to be more stable in crystalline TClHCl physical blends with PVP or PEC than in amorphous solid dispersions at the same vitamin:polymer ratio (Figure 4A), which is further evidence that amorphous thiamine is more labile and therefore less stable than crystalline thiamine; of note, differences in thiamine stability were also found in different amorphous dispersions (Figure 3). Differences in chemical stability in different amorphous matrices have been attributed to differences in T_g_, wherein a lower T_g_ is generally associated with decreased stability due to increased molecular mobility, especially when the storage temperature is above the T_g_ [14,22]. This has been shown to affect thiamine stability due to the effect of the excipient on resulting solid dispersion T_g_ [19]. Additionally, the magnitude of difference between storage temperature and T_g_ (T−T_g_) also affects molecular mobility: as T−T_g_ increases, molecular mobility increases, which usually correlates to decreased stability in terms of both physical change and chemical reactivity [22,23].

Immediately following lyophilization, the T_g_ values of samples containing ≤80% PVP were below 60 °C, further decreasing as the amount of PVP decreased because the T_g_ of the polymer was greater than that of amorphous thiamine (Table 2). The presence of the PVP polymer inhibited thiamine crystallization (Figure 2A), even above the T_g_ for some of the vitamin:polymer ratios studied [15]. Even when the samples were exposed to 11% RH, which increased the moisture content of the samples and thereby decreased the T_g_, PVP still inhibited thiamine crystallization. The scope of this study therefore included formulations and storage conditions that enabled comparisons of thiamine degradation in the amorphous state in both glassy and supercooled liquid matrices. For example, the initial T_g_ of the 5TClHCl:95PVP solid dispersion was above 60 °C, the highest temperature used in this study, while the T_g_ following equilibration at 11% RH and 60 °C was lowered to 47 °C, indicating the change of the solid dispersion to the supercooled liquid state (Table 3). This same dispersion type equilibrated at 11% RH and 30 °C had a T_g_ of 54 °C and therefore remained in the glassy state (Table 3). Increasing storage temperature increased thiamine degradation in these dispersions: those stored at 60 °C had the most thiamine degradation after 56 days (76% thiamine remaining), followed by those stored at 50 °C (88% thiamine remaining) (Figure 4C). Less thiamine degraded in the samples stored at temperatures below the T_g_, with 94% thiamine remaining in the samples stored at 30 °C at the end of the study. These results are consistent with the general theory that the glassy state is more stable than the supercooled liquid state of a matrix and the increase of T−T_g_ further decreases stability [19,22].

However, despite having the lowest T_g_, thiamine was the most chemically stable in the amorphous 40TClHCl:60PVP dispersions when comparing the samples containing ≤30% TClHCl, and thiamine stability further decreased as the vitamin:polymer ration decreased (Figure 3). In fact, the dispersion with the highest T_g_ (1TClHCl:99PVP) (Table 2) had the highest rate of thiamine degradation (Table 1). This indicates that factors other than T_g_ are presumably influencing chemical stability behaviors of amorphous thiamine. Generally, the correlation between sample T_g_ and chemical stability can only be analyzed in identical samples. If the samples are not identical to each other (e.g., samples prepared with different vitamin:polymer ratios or using a different polymer), other factors, including intermolecular interactions and contact surface area between compounds, could be the major determinant for chemical stability, rather than T_g_. Numerous publications have shown that a higher T_g_ does not always result in increased chemical stability [24,25,26,27]. For example, Bell and Hageman [24] investigated aspartame stability in two different PVP polymers possessing different T_g_ values in systems with similar moisture contents and water activities (*a_w_*). Although the PVP T_g_ values were different, no difference was found in degradation rate constants for aspartame [24]. Moreover, a recent study has shown that ascorbic acid stability was actually higher in the samples with lower T_g_ values than those with higher T_g_ values, wherein chemical degradation occurred in the glassy state [27]. Therefore, the T_g_ values of the solid dispersions in this study were not considered to be the driving factor for differences in the chemical stability of thiamine across formulations, but T−T_g_ did correlate with the effect of storage temperature on thiamine stability in the same matrix.

### 2.3. Effect of Polymer Type on Thiamine Degradation in Amorphous Solid Dispersions

In addition to differences in thiamine chemical stability between amorphous and crystalline matrices, as well as differences in stability due to storage conditions, the type of polymer present in the amorphous matrix was also found to have a significant effect on thiamine stability in the amorphous state (Figure 4). Significantly more (*p* < 0.05) thiamine degraded in the presence of PVP than when formulated with PEC in the solid dispersions. A series of studies was undertaken to better understand why thiamine was more stable in dispersions with PEC than in dispersions with PVP, encompassing pH, moisture content, T_g_, and intermolecular interactions.

Sample pH has been reported to be an important factor affecting thiamine stability in solutions [12,18]. As shown in Table 4, the pH levels of pre-lyophilized solutions of 5TClHCl:95PVP and 5TClHCl:95PEC were 3.8 and 3.5, respectively, which are both below the food-relevant pK_a_ of thiamine (4.8). A speciation plot of thiamine illustrates that the more stable protonated (pyrimidine N1) species was dominant at both of these pH values [12]. Although the fraction of protonated species was slightly higher in 5TClHCl:95PEC solutions (~95%) than in 5TClHCl:95PVP solutions (~91%), this slight difference in fraction of protonated species was not likely to have caused the substantial difference in chemical stability between PEC- and PVP-based dispersions, with the dispersions containing 96% and 76% thiamine, respectively, following 56 days of storage at 11% RH and 60 °C (Figure 4). Additionally, it has been reported that pre-lyophilized solution pH does not always correlate to pH of the amorphous solid dispersion [28]; thus, pH was not considered the major factor influencing the differences in thiamine stability between PEC- and PVP-based dispersions.

**Figure 5:**
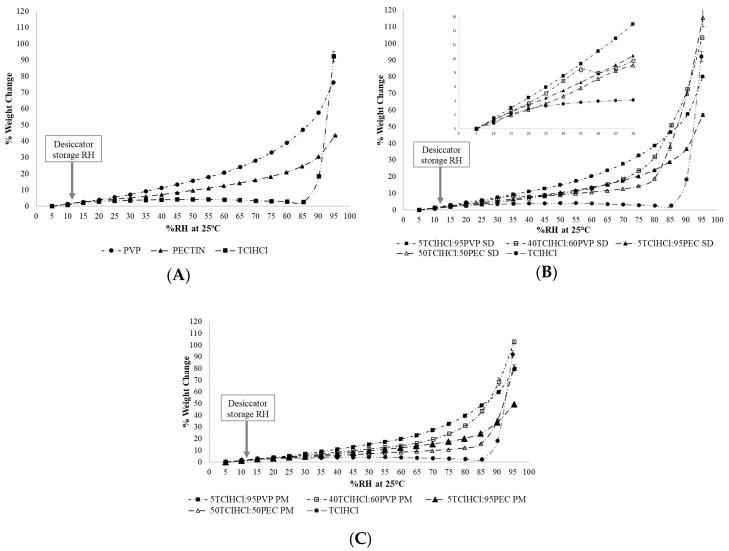
Moisture sorption profiles of (**A**) PVP, PEC, and TClHCl at 25 °C; (**B**) TClHCl, 40TClHCl:60PVP, 5TClHCl:95PVP, 50TClHCl:50PEC, and 5TClHCl:95PEC solid dispersions (SDs) at 25 °C, with insert graph highlighting initial moisture sorption differences between SDs from 5–50% RH; and (**C**) 40TClHCl:60PVP, 5TClHCl:95PVP, 50TClHCl:50PEC, and 5TClHCl:95PEC physical mixtures (PMs) at 25 °C. The arrows in the profiles identify 11% RH, the desiccator storage RH, indicating that the moisture contents of all samples were similar at the storage RH.

Moisture sorption isotherms of individual ingredients, TClHCl:polymer solid dispersions, and physical mixtures of TClHCl with polymers generated at 25 °C are compared in Figure 5A–C. When considering the individual ingredients, significantly more moisture was absorbed by PVP when compared to PEC, especially above 30% RH, and the difference in sorbed moisture increased as RH increased (Figure 5A). Solid vitamin:polymer dispersions followed the same general trend as individual polymers for moisture sorption, wherein PVP dispersions sorbed significantly more moisture than PEC dispersions, regardless of the polymer amount used (Figure 5B). This can be attributed to the more hygroscopic nature of PVP compared to PEC. At low-RH conditions such as those used in this study (11% RH), however, no significant differences in moisture sorption between the solid dispersions were found. For example, significantly more thiamine (20% more) degraded in 5TClHCl:95PVP dispersions than 5TClHCl:95PEC dispersions at 11% RH and 60 °C after 56 days of storage, but no significant difference in the amount of moisture gained by the dispersions of 5TClHCl:95PVP and 5TClHCl:95PEC at 11% RH was found. However, moisture sorption profiles were conducted at 25 °C, so the increased temperature should also be considered. Based on the moisture sorption findings in this study, no direct relation was found between the chemical stability of thiamine and hygroscopicity of polymers/dispersions at the low-RH condition studied.

Different polymers have different T_g_ values, with PVP having a higher T_g_ (134 °C) than PEC (90 °C) [15]. Based on these values, the T_g_ values of PVP dispersions were expected to be higher than T_g_ values of PEC dispersions of the same vitamin:polymer ratios prior to storage. However, TClHCl degraded more in PVP dispersions than in PEC dispersions (Figure 4A). Additionally, more thiamine degraded in PVP dispersions with the highest T_g_ values (and highest proportions of polymer) (Table 1 and Table 2; Figure 3A). Therefore, differences in T_g_ were not considered to be the reason for the differences in the chemical stability of TClHCl in these solid dispersions.

**Figure 6:**
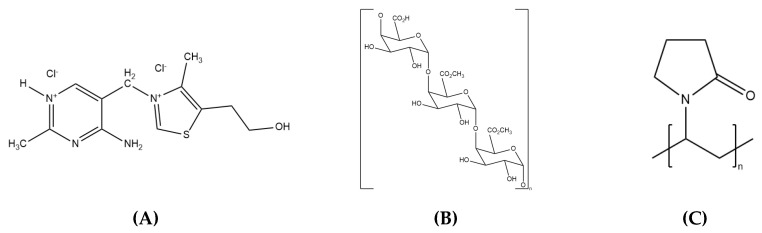
Chemical structures of (**A**) thiamine, (**B**) pectin, and (**C**) PVP.

Chemical structures and properties of polymers and TClHCl were also considered for their relations to chemical stability. Structures of thiamine and the polymers (Figure 6) were extensively reviewed by Arioglu-Tuncil, Bhardwaj, Taylor, and Mauer [15] in terms of functional groups (and hydrogen bond acceptor group strengths based on the p*K*_BHX_ scale), which may be involved in hydrogen bonding. Thiamine is comprised of a thiazole and a pyrimidine ring, which are connected to each other via a methylene bridge. Free hydroxyl and NH_2_ groups of TClHCl can act as both hydrogen bond donors (HBDs) and acceptors (HBAs). In addition, the nitrogen atoms on the pyrimidine ring can interact via hydrogen bonding as weak HBAs [15]. PEC possesses a variety of functional groups, including hydroxyl, carboxylic acid, ether, and ester groups, that can all participate in hydrogen bonding. On the other hand, the only functional group on PVP able to hydrogen bond is the amide carbonyl group, which acts as a strong HBA [15].

This difference in hydrogen bonding potential between PEC and PVP with TClHCl has been shown previously using Fourier transform infrared spectroscopy (FTIR), as related to amorphization capabilities of the polymers [15]. Briefly, peak shifts to lower wavenumbers in FTIR spectra of vitamin:polymer dispersions compared to polymers alone indicate stronger/more extensive intermolecular interactions. In the mentioned study, TClHCl:PEC dispersions were found to have peak shifts up to 148 cm^−1^ in the hydroxyl region, indicating very strong hydrogen bonding interactions between PEC and TClHCl [15]. Moreover, TClHCl:PEC dispersions were also found to have peak shifts up to 18 cm^−1^ in the carbonyl region compared to only 1 cm^−1^ peak shifts in the TClHCl:PVP dispersions, indicating comparatively stronger intermolecular interactions between TClHCl and PEC than between TClHCl and PVP [15]. TClHCl also has the potential for ionic interaction with the carboxylic group in PEC. The stronger hydrogen bonding and/or ionic interactions in TClHCl:PEC dispersions compared to PVP led to greater physical stability of amorphous thiamine shown in that study [15]. The intermolecular interactions between thiamine and polymers in the solid dispersions were therefore also proposed as the explanation for the greater chemical stability of TClHCl:PEC amorphous solid dispersions compared to TClHCl:PVP dispersions, wherein PEC protected thiamine against chemical degradation by restricting the molecular mobility of TClHCl (higher molecular mobility resulted in an increased degradation rate). A similar observation was also reported by Ismail and Mauer [29], who found that stronger intermolecular interactions between PVP and ascorbic acid than between PVP and sodium ascorbate were likely a contributing factor for the higher stability of ascorbic acid in these PVP-based amorphous solid dispersions. Due to lack of interactions between TClHCl and PVP in the amorphous solid dispersions in this study, thiamine was significantly less stable in PVP dispersions than in PEC dispersions that had more vitamin–polymer intermolecular interactions.

### 2.4. Chemical Stability of Thiamine Related to Polymer Proportion in a Solid Dispersion

Thiamine had decreased stability in solid dispersions containing higher proportions of PVP compared to those with less polymer. For example, solid dispersions containing 20TClHCl:80PVP, with a t_90_ of 70 days, were more stable than those composed of 1TClHCl:99PVP, which had a t_90_ of 17 days (Table 1). This finding is consistent with results from a study of ascorbic acid in which the chemical stability of amorphous ascorbic acid in PVP dispersions was found to decrease during storage at 11% RH and 60 °C as the relative proportion of PVP in the dispersions increased [27]. This observation was attributed to a kinetic model developed by Waterman et al. [30] in which solid state degradation is directly related to the relationship between the drug and the excipient. This kinetic model proposes that if the numbers of drug and excipient particles are comparable to each other, drug concentration does not play a role in its degradation rate. In contrast, if the excipient particles are found in excess in the system, the drug-to-excipient ratio has an effect on the drug degradation rate due to greater surface area of contact between the drug and the excipient (degradation rate increases with increasing contact surface area) [30]. The increased surface area, in turn, enables a greater extent of drug–excipient interactions, which are detrimental to chemical stability. This mechanism can be applied to the PVP-based solid dispersion chemical stability data, in which thiamine degradation increased in TClHCl:PVP dispersions as the number of PVP molecules increased relative to that of TClHCl. The increase in PVP content occurs concurrently with more thiamine–polymer interactions, at the expense of thiamine–thiamine interactions, where such interactions are clearly detrimental to thiamine chemical stability. Thus, the chemical stability of thiamine in systems containing different proportions of thiamine to polymer was presumably largely dictated by the extent of molecular interactions between thiamine and polymer.

## 3. Materials and Methods

### 3.1. Materials

Thiamine chloride hydrochloride (TClHCl), C_12_H_17_ClN_4_OS • HCl, pectin (PEC, from citrus peel with a ~61% degree of esterification), and poly(vinylpyrrolidone) (PVP, MW 40,000) were obtained from Sigma-Aldrich Inc. (St. Louis, MO, USA). The two polymers were chosen based on previous work [15] in which PEC was found to be the best crystallization inhibitor for TClHCl while PVP was found to be the poorest crystallization inhibitor. These polymers were chosen to represent a variety of polymers used in the food and pharmaceutical industries. They were also chosen due to their chemical and physical properties, including propensity for hydrogen bonding and ionic interactions with TClHCl, hygroscopicity, and T_g_. Additionally, PVP is commonly used to investigate amorphous food ingredient stability, and PEC is commonly found in foods [24,27]. Specific RH conditions (reported here at 25 °C) were created in desiccators using saturated salt solutions of lithium chloride (LiCl, 11% RH) (EMD Millipore, Billerica, MA, USA). HPLC grade trifluoroacetic acid (TFA) was obtained from Sigma-Aldrich Inc. (St. Louis, MO, USA), and acetonitrile was purchased from Fisher Scientific Co., LLC (Pittsburgh, PA, USA). Deionized and purified water was used in the study, prepared using a Barnstead E-Pure ultrapure water purification system (ThermoScientific, Waltham, MA, USA) with a resistivity of ~17.5 MΩ·cm.

### 3.2. Preparations of TClHCl Solid Dispersions via Lyophilization

TClHCl solid dispersions were prepared using a previously established lyophilization method [15]. Amorphization by lyophilization was chosen to avoid exposure to heat, which may have degraded the thiamine during the amorphization process. Samples were prepared in triplicate using a range of vitamin:polymer mass ratios, ranging from 1 to 90% TClHCl with PVP and 5 to 50% for TClHCl with PEC (Table 5). A total of 100 mg of solids containing the desired vitamin:polymer ratio was dissolved in 10 mL of deionized water in a 20 mL amber glass vial and mixed with a Roto-Shake Genie SI-1100 (Scientific Industries, Inc., Bohemia, NY, USA) for 10 min. The solutions were then frozen overnight at −20 °C prior to lyophilization. After removal from the freezer, samples were placed into a VirTis Genesis 25ES shelf freeze dryer (SP Scientific, Stone Ridge, NY, USA) and frozen for 6 h at −40 °C and 300 mTorr. For the primary drying step, high vacuum was applied (150 mTorr) at −40 °C for 24 h to remove the bulk of water via sublimation. Secondary drying was then achieved by holding the samples for 9 h each at 10 °C intervals from −40 to 20 °C. Lastly, the samples were held for 6 h at 25 °C at 300 mTorr. Upon completion of the cycle, the lyophilized samples were immediately placed into RH-controlled desiccators. Physical blends of crystalline TClHCl with polymers (5TClHCl:95polymer) were also prepared by weighting each ingredient separately, followed by simply mixing in amber vials.

### 3.3. Storage Treatments

The following temperature and RH conditions were chosen as storage treatments: 11% RH and 30, 40, 50, and 60 °C. Increasing temperature conditions at 11% RH were chosen in order to enable reaction kinetics calculations of thiamine degradation, similar to studies by Voelker, Miller, Running, Taylor, and Mauer [12]. RH was controlled by using saturated salt solutions in desiccators, with the water activity of the salt solutions verified by measurement using an AquaLab 4TE water activity meter (Decagon Devices Inc., Pullman, WA, USA). To control the temperature, the desiccators were placed into water-jacketed incubators (Forma Scientific, Inc., Marietta, OH, USA). Samples were stored for up to 8 weeks, with a subset removed biweekly for HPLC analysis and then discarded after analysis.

### 3.4. Powder X-Ray Diffraction (PXRD)

Powder X-ray diffractograms of the starting ingredients and dispersions at set storage intervals were obtained using a Rigaku SmartLab diffractometer (Rigaku Americas, TX, USA) equipped with a Cu-Kα radiation source and D/teX Ultra detector. Samples were scanned from 5 to 40° 2 θ with an increment of 0.02° and a rate of 4° per min. Structural distinction between amorphous and crystalline TClHCl was determined based on the PXRD patterns, in which samples exhibiting a diffuse halo were considered to be PXRD amorphous, and those containing sharp peaks in their diffractograms above a signal-to-noise ratio of 3 were considered to be crystalline.

### 3.5. Chemical Stability Determination with HPLC

TClHCl was quantified in the absence and presence of polymers throughout the study using a Waters 2690SM HPLC with a Waters XSelect HSS T3 (3.5 µm, 4.6 × 100 mm) column and a Waters 2996 photodiode array detector (Waters Corp., Milford, MA, USA). Prior to each analysis, standard curves of TClHCl at a concentration range from 0.005 to 1 mg/mL were prepared (R^2^ = 0.9997−1.0000). Samples were diluted with solvent and filtered through a 0.2 µm syringe filter. The mobile phase containing solvent A (acetonitrile) and solvent B (water and 0.1% TFA) was used with the following gradient procedure adapted from Xia et al. [31]: 0/100 at 0 min (immediate), 3/97 at 4 min (linear), 10/90 at 6 min (linear), 0/100 at 10 min (linear), and 0/100 from 10 to 15 min (immediate), for a total chromatographic run time of 15 min. The flow rate was 1 mL/min, and the samples were scanned between 235–400 nm. Integration was conducted at 247 nm using MassLynx software (V4.1, Waters Corp., Milford, MA, USA).

### 3.6. Water Content and Moisture Sorption

An SPSx-1 μ Dynamic Vapor Sorption Analyzer (Project Messtechnik, Ulm, Germany) was used to generate moisture sorption profiles of the individual ingredients, solid dispersions, and physical blends at 25 °C. Approximately 100–300 mg of each sample was weighed into the 18 mm aluminum sample pan (Mettler Toledo, Columbus, OH, USA) in a 24-ring sample holder. Data were collected using an equilibrium criterion of a weight change of 0.001% in 30 min and a maximum step time of 12 h. Samples were exposed to 0% RH for 12 h and then analyzed from 5 to 95% with a 5% RH step size. The moisture content of select samples was determined immediately following freeze drying using a method adapted from Arabshahi and Lund [20]. Briefly, solid dispersions were weighed into aluminum pans and placed in the vacuum oven. Then, the samples were exposed to 45 °C under vacuum for 48 h. To limit degradation of thiamine, the use of a higher temperature was avoided. Moisture content (%) was calculated on a wet weight basis using the following formula [32]:

(1) % Moisture (wtwt)=wt of wet sample−wt of dry samplewt of wet sample×100

### 3.7. Determination of Glass Transition Temperature (T_g_) by Differential Scanning Calorimetry (DSC)

Thermal analyses of the starting ingredients and lyophiles were carried out using a Discovery DSC equipped with a refrigerated cooling accessory (TA Instruments, New Castle, DE, USA). Nitrogen (50 mL/min) was used as the purge gas. Samples were weighed (7–12 mg) and hermetically sealed into Tzero pans (TA Instruments, New Castle, DE, USA). To determine the T_g_ values of the samples, the samples were heated from −20 °C to a temperature 20–30 °C higher than the expected T_g_ at a rate of 20 °C/min. Samples were then cooled to −20 °C at 10 °C/min, followed by heating to 150 °C at a rate of 20 °C/min. The onset glass transition temperature of the second heating step was reported as T_g_ unless otherwise stated.

To determine onset T_g_’ of the frozen samples prior to lyophilization (for determining effective primary drying temperature during lyophilization), approximately 25 μL of solutions were pipetted into Tzero pans and sealed. Solutions were first cooled to −80 °C and held for 5 min at this temperature before being heated to 0 °C at a rate of 10 °C/min. TRIOS software was used to determine the T_g_ or T_g_’ from the scans (TA Instruments, New Castle, DE, USA).

### 3.8. Reaction Kinetics Calculations

Chemical degradation of thiamine has been reported to follow first-order reaction kinetics [17,18,33]. Therefore, reaction rate constants were calculated based on the following equation:

(2) $$\ln\frac{x\;}{x_{0}}=-kt$$

where *x* is thiamine concentration at time t (days), *x*_0_ is the initial concentration of thiamine, and *k* is defined as a reaction rate constant (days^−1^). The following equation was used for shelf life estimation to calculate t_90_, which is defined as the time where 90% of the initial concentration of thiamine remains (or 10% has degraded):

(3) $$t_{90}=\frac{\ln\left(0.9\right)}{-k}$$

where *k* is the reaction rate constant (days^−1^).

### 3.9. Statistical Analysis

The HPLC, water content, and DSC analyses were performed in triplicate, and data are presented as mean ± standard deviation. SAS Software Version 9.4 (SAS Institute, Cary, NC, USA) was used to conduct the statistical analyses. Analysis of variance (ANOVA) was performed at α = 0.05 significance level to determine differences among the samples and controls. Tukey’s multiple comparison test (α = 0.05) was used to test whether samples were statistically different.

## 4. Conclusions

Thiamine was stable against degradation in the crystalline state, and the chemical stability of crystalline TClHCl was unaffected by the presence of a polymer in physical blends in the environmental conditions studied. In contrast, significantly more thiamine degraded when in the amorphous form, which was also highly affected by type and proportion of polymer in the amorphous solid dispersion as well as by the storage conditions. Amorphous thiamine was more stable in dispersions made using pectin than those using PVP at all storage treatments. No direct correlation was found between chemical stability of thiamine and hygroscopicity or T_g_ of solid dispersions containing different polymers. The formation of intermolecular interactions between TClHCl and polymer decreased thiamine degradation: the greater tendency for hydrogen bonding and ionic interactions between TClHCl and pectin than between TClHCl and PVP led to thiamine in TClHCl:PEC dispersions being more stable than that in PVP dispersions. Thiamine degradation was significantly higher in samples containing lower concentrations of thiamine relative to PVP than in those with less PVP, which was mainly governed by the extent of surface area of molecular contact between TClHCl and PVP. In summary, the solid-state form of TClHCl (amorphous vs. crystalline), polymer type (e.g., pectin vs. PVP), relative proportions of vitamin and polymer, polymer structural properties, and storage conditions were all important factors influencing the chemical stability of thiamine, with intermolecular interactions between TClHCl and the polymer and vitamin:polymer ratios playing significant roles in the stability of amorphous thiamine. Taken with our previous work on the amorphization of thiamine using polymers, these two studies showcase the physical and chemical stability of amorphous thiamine–polymer systems, which is necessary for understanding thiamine delivery in foods or dietary supplements in which amorphous thiamine is likely to exist. All factors need to be considered and carefully selected for improving the stability of thiamine in foods and supplements formulated with TClHCl. The effect of molecular structure of the polymers and the intermolecular interactions between the polymer and TClHCl on the chemical stability of thiamine can be used to predict thiamine stability in a variety of food and dietary supplement systems in which thiamine exists in the amorphous state. The fact that decreasing vitamin proportion in an amorphous matrix increased the degradation rate of the vitamin is relevant for low- and intermediate-moisture foods and supplements, wherein vitamins are present in low concentrations and the degradation of the sulfur-containing thiamine may alter both nutrition and sensory profiles of the products.

## Figures and Tables

**Table 1:** Rate constants and t_90_ values of thiamine degradation in solid dispersions prepared with different proportions of PVP, upon storage at 11% RH and 60 °C.

Sample	k_obs_ (day^−1^)	R^2^	t_90_ (days)
1TClHCl:99PVP	0.0062	0.96	17
3TClHCl:97PVP	0.0047	0.95	22
5TClHCl:95PVP	0.0038	0.95	28
7TClHCl:93PVP	0.0032	0.93	33
10TClHCl:90PVP	0.0025	0.95	42
20TClHCl:80PVP	0.0015	0.92	70

k_obs_: observed reaction rate constant; R^2^: indicates linear correlation of regressions used for k_obs_ calculations; t_90_: time when 90% of the initial concentration of thiamine remained.

**Table 2:** Onset glass transition temperatures (T_g_) and moisture contents of amorphous solid dispersions with different TClHCl:PVP ratios immediately following lyophilization.

Sample	Onset T_g_ (°C)	% Moisture Content (wb)
1TClHCl:99 PVP SD	61 ± 2 ^A^	2.1 ± 0.5 ^a^
5TClHCl:95 PVP SD	61 ± 2 ^A^	1.8 ± 0.1 ^a^
20TClHCl:80 PVP SD	54 ± 7 ^BA^	1.3 ± 0.7 ^a^
30TClHCl:70 PVP SD	55 ± 4 ^BA^	1.39 ± 0.07 ^a^
40TClHCl:60 PVP SD	42 ± 5 ^B^	0.8 ± 0.2 ^a^

Superscript letters indicate statistical significance within each column.

**Table 3:** Onset glass transition temperatures (T_g_) of amorphous solid dispersions equilibrated at identified conditions.

Sample	Storage Condition	Onset T_g_ (°C)
5TClHCl: 95PVP SD	11% RH and 30 °C	53.6 ± 0.1 ^A^
5TClHCl:95PVP SD	11% RH and 60 °C	47 ± 1 ^B^

RH: relative humidity; Superscript letters indicate statistical significance.

**Table 4:** The pH values of TClHCl solutions at 25 °C prior to lyophilization.

Pre-Lyophilized Solution Composition (100 mg Solids/10 mL Water)	pH (at 25 °C)
1TClHCl:99PVP	3.94 ± 0.01 ^B^
3TClHCl:97PVP	3.905 ± 0.007 ^B^
5TClHCl:95PVP	3.800 ± 0.004 ^BC^
7TClHCl:93PVP	3.73 ± 0.06 ^C^
10TClHCl:90PVP	3.71 ± 0.01 ^CD^
20TClHCl:80PVP	3.54 ± 0 ^E^
30TClHCl:70PVP	3.47 ± 0.03 ^EFG^
40TClHCl:60PVP	3.44 ± 0.02 ^EFGH^
50TClHCl:50PVP	3.38 ± 0.01 ^FGHI^
60TClHCl:40PVP	3.345 ± 0.007 ^GHIJ^
70TClHCl:30PVP	3.315 ± 0.007 ^HIJ^
80TClHCl:20PVP	3.27 ± 0.01 ^IJ^
90TClHCl:10PVP	3.25 ± 0.02 ^IJ^
100TClHCl	3.23 ± 0.02 ^J^
100PVP	4.1 ± 0.1 ^A^
5TClHCl:95PEC	3.525 ± 0.007 ^EF^
100PEC	3.58 ± 0 ^ED^
50TClHCl:50PEC	3.31 ± 0 ^HIJ^

Superscript letters indicate statistical significance.

**Table 5:** Formulations of all TClHCl solid dispersions investigated in this study.

% TClHCl in Formulation	% PVP in Formulation	% Pectin in Formulation
-	100%	-
1%	99%	-
3%	97%	-
5%	95%	-
7%	93%	-
10%	90%	-
20%	80%	-
30%	70%	-
40%	60%	-
50%	50%	-
60%	40%	-
70%	30%	-
80%	20%	-
90%	10%	-
100%	-	-
-	-	100%
5%	-	95%
50%	-	50%

## Abbreviations

DSC	Differential scanning calorimetry
FD	Freeze dried
FTIR	Fourier transform infrared spectroscopy
HBA	Hydrogen bond acceptor
HBD	Hydrogen bond donor
HPLC	High-performance liquid chromatography
PEC	Pectin
PM	Physical mixture
PVP	Poly(vinylpyrrolidone)
PXRD	Powder x-ray diffraction
RH	Relative humidity
SD	Solid dispersion
TClHCl	Thiamine chloride hydrochloride
TFA	Trifluoroacetic acid
T_g_	Glass transition temperature
